# I love me - Body Image Peer Mentor, Support and Leadership Program

**DOI:** 10.1186/2050-2974-3-S1-O18

**Published:** 2015-11-23

**Authors:** Amanda Dearden, Alison Lee

**Affiliations:** 1 Isis- the Eating Issues Centre Inc, Australia

## 

Isis- The Eating Issues Centre Inc. School Based Prevention and Early Intervention Evaluation

Isis- ‘I Love Me’, is an interactive program promoting a whole of school approach to promoting positive body culture using creative, interactive age appropriate activities and discussions. Quantitative and Qualitative evaluation of 6 pilot schools will be presented alongside learnings and recommendations.

Pre and post measures of students were evaluated (n= 110). 59 year ten, 38 year nine, 2 year eleven and 2 year twelve students participated comprised of females 53.6%, males 29.1% with an age range from 13 to 17. Control group included 36 and 74 completed the pilot program.

Qualitative measures indicated increase in students self-reported knowledge of body image; confidence in referring, ability to contribute to improved body image in others, increased satisfaction and acceptance of own body. Quantitative results include:

• The Sociocultural Attitudes scale showed a significant reduction in media influence across all of the subscales after Isis training, including significant decrease in body surveillance.

• The Empowerment scale showed an improvement in self-esteem, increased capability of achieving outcomes as a community, increased optimism and sense of control over the future.

